# Hypoglycemia Associated With Drug–Drug Interactions in Patients With Type 2 Diabetes Mellitus Using Dipeptidylpeptidase-4 Inhibitors

**DOI:** 10.3389/fphar.2021.570835

**Published:** 2021-04-15

**Authors:** Chin-Ying Ray, Victor Chien-Chia Wu, Chun-Li Wang, Hui-Tzu Tu, Yu-Tung Huang, Chang-Fu Kuo, Shang-Hung Chang

**Affiliations:** ^1^ Department of Pharmacy, Chang Gung Memorial Hospital, Linkou Medical Center, Taoyuan City, Taiwan; ^2^ Division of Cardiology, Chang Gung Memorial Hospital, Linkou Medical Center, Taoyuan City, Taiwan; ^3^ College of Medicine, Chang Gung University, Taoyuan City, Taiwan; ^4^ Center for Big Data Analytics and Statistics, Chang Gung Memorial Hospital, Linkou Medical Center, Taoyuan City, Taiwan; ^5^ Division of Rheumatology, Allergy and Immunology, Department of Internal Medicine, Chang Gung Memorial Hospital, Linkou Medical Center, Taoyuan City, Taiwan; ^6^ Division of Rheumatology, Orthopaedics and Dermatology, School of Medicine, University of Nottingham, Nottingham, United Kingdom

**Keywords:** dipeptidylpeptidase-4 inhibitors, drug-drug interaction, drug safety, hypoglycemia, diabetes mellifus

## Abstract

**Background:** Dipeptidylpeptidase-4 inhibitors (DPP-4i′s) are considered to be safe for patients with type 2 diabetes mellitus (T2DM). However, little is known about drug–drug interactions between DPP-4i′s and concurrent medications.

**Methods:** Data on patients using DPP-4i′s for T2DM during 2011–2017 were retrieved from Chang Gung Research database provided by Chang Gung Memorial Hospital. Patients were excluded if they were aged <30 years or >90 years; had incomplete demographic data; had insulinoma; or had records of concomitant insulin use. A generalized estimating equation–based Poisson model was employed for statistical analysis. The primary outcome was hypoglycemia events.

**Results:** We retrieved data on a total of 97,227 patients using DPP-4i′s. After patients were excluded according to the mentioned criteria, the remaining 77,047 DPP-4i users were studied (mean age 64 ± 12 years, men 54.4%). The most common medications coprescribed with DPP4is over all person-quarters were acetaminophen, simvastatin, fluvastatin, and colchicine (all >20,000 person-quarters). The combinations of a DPP-4i with bumetanide, captopril, colchicine, acetaminophen, cotrimoxazole, and pantoprazole were associated with an increased risk of hypoglycemia. Compared with the ratios observed for person-quarters of DPP-4i use alone (reference category), the adjusted prevalence ratios per 100 person-years of hypoglycemia for person-quarters of DPP-4i use in combination with bumetanide, captopril, colchicine, acetaminophen, cotrimoxazole, and pantoprazole were 2.44 (95% confidence interval [CI], 1.78–3.36), 2.97 (95% CI, 2.26–3.90), 1.87 (95% CI, 1.44–2.42), 2.83 (95% CI, 2.44–3.29), 2.27 (95% CI, 1.27–4.04), and 3.03 (95% CI, 1.96–4.68), respectively.

**Conclusion:** Among patients taking DPP-4i′s for T2DM, concurrent use of such inhibitors with bumetanide, captopril, acetaminophen, and pantoprazole was associated with an increased risk of hypoglycemia compared with the use of DPP-4i′s alone. Physicians prescribing DPP-4i′s should consider the potential risks associated with their concomitant use with other drugs.

## Introduction

Therapeutic armamentaria for the treatment of type 2 diabetes mellitus (T2DM) have been increasingly diversified in mechanism of actions in recent decades. Dipeptidylpeptidase-4 inhibitors (DPP-4i′s), also known as gliptins, are among the newest categories of antidiabetic medications (ADMs) (Idris and Donnelly, 2007). DPP-4i′s were developed to improve glucose control through the increase in incretin levels (GLP-1 and GIP) that which inhibit glucagon release and produce more insulin only when it is needed, with benefit of no weight gain and rare hypoglycemic events (Scheen, 2005; Tella and Rendell, 2015). Since T2DM patients often have comorbid conditions such as cardiovascular diseases requiring the use of additional antihypertensive or antihyperlipidemic agents, it is important to ascertain that the risks of drug–drug interactions when multiple medications are prescribed together (Scheen, 2010; Cosentino et al., 2019).

DPP-4i′s that have been brought to the market comprise sitagliptin, saxagliptin, linagliptin, vildagliptin, and alogliptin, existing either as single agents or in fixed-dose combined formulations with metformin (Ahrén and Foley, 2016; Nishimura et al., 2019). Preclinical studies with DPP-4i′s pharmacokinetic interactions have described with some of commonly coprescribed medications, including diuretics, angiotensin-converting enzyme (ACE) inhibitors, calcium channel blockers, statins, antibiotics, and proton pump inhibitors, with DPP-4i′s (Kim et al., 2006; Ayalasomayajula et al., 2007; Chu et al., 2007; He et al., 2008; Patel et al., 2011; Patel et al., 2011; Arulmozhiraja et al., 2016; May and Schindler, 2016). The knowledge of these scientific hypotheses in the laboratory however, cannot supplant clinical scenarios where drug-drug interactions may be far more complicated then what is known from translational medicine. Limited data exists in real-world situations where these drug-drug interactions are examined in large-scale patients using DPP-4i′s. Accordingly, we conducted this study to investigate the drug–drug interactions between DPP-4i′s and commonly concurrent medications, measure the frequency of such coprescriptions, and describe the associated risk of hypoglycemia in the coprescribed medicaitons.

## Methods

### Data Source

In this retrospective cohort study, patient data were obtained from the largest health care provider in Taiwan, the Chang Gung Memorial Hospital system, which comprises three major teaching hospitals and four tertiary-care medical centers (Tsai et al., 2017; Wang et al., 2018; Wang et al., 2019; Wang et al., 2019). The health care provider has more than 10,000 beds and admits more than 280,000 patients, servicing approximately one-tenth of the Taiwanese population each year. The hospital identification number of each patient was encrypted and deidentified to protect their privacy. Therefore, informed consent was waived for this study. The diagnosis and laboratory data could be linked and continuously monitored using consistent data encryption. To examine ADM use among patients with T2DM, we retrieved data for a population-based panel of patients with T2DM between January 1, 2011, and December 31, 2017, from the Chang Gung Research Database (CGRD) provided by Chang Gung Memorial Hospital. The Institutional Review Board of Chang Gung Memorial Hospital approved the study protocol (IRB No. 201802084B1).

### Study Design

**FIGURE 1:**
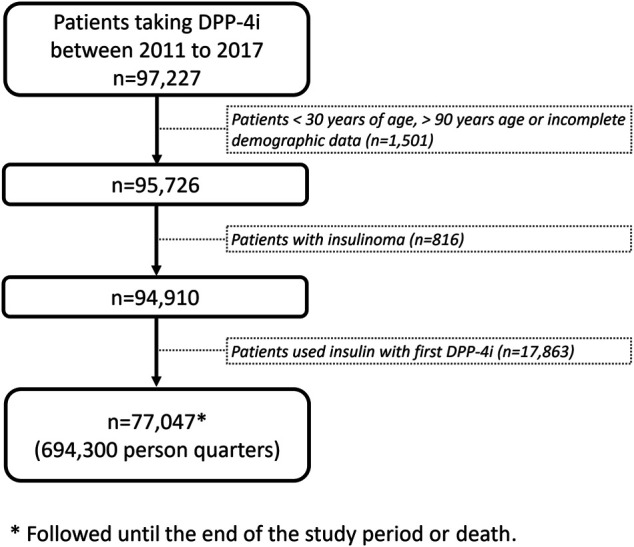
Study design and flowchart for patient enrollment.

We designed a panel study for patients with T2DM who received DPP-4i prescriptions between 2011 and 2017, We followed the patients until death or until May 31, 2018, the end of the study period. Follow-up data for each included patient were analyzed at the person-quarter level, which served as the analytic unit. Information on comedications and outcomes was collected by each observed person-quarter. We excluded patients who were aged <30 or >90 years; had incomplete demographic data; received a diagnosis of insulinoma; or used insulin in conjunction with a DPP-4i (Figure 1).

### Selected Drugs for the Study of Drug–Drug Interactions with DPP-4i′s.

After reviewing the medical literature for studies describing drug–drug interactions between commonly prescribed medications and DPP-4i′s, we selected the following medications for investigation: bumetanide (a diuretic), captopril and fosinopril (ACE inhibitors), verapamil (a calcium channel blocker), simvastatin and fluvastatin (statins), gemfibrozil (a fibrate), duloxetine (an anxiolytic agent), sulfinpyrazone and colchicine (uric acid–lowering agents), acetaminophen (an analgesic agent), cotrimoxazole (an antibiotic agent), and pantoprazole (a proton pump inhibitor).

### Follow-up Periods and Person-Quarters

In this study, each calendar year was partitioned into four quarters for each patient and each year after the first DPP-4i prescription. The analytic unit was one person-quarter. Person-quarters were used because medications for chronic illnesses were refilled after a maximum of 3 months according to the Taiwan National Health Insurance reimbursement policy, as previously described (Chang et al., 2017; Wang et al., 2019 Aug 6). Accordingly, medications and covariates were assessed for each person-quarter, which simplified the assessment of the complex prescription pattern of DPP-4i′s and multiple drugs. Person-quarters exposed to DPP-4i′s with or without concurrent medications were identified. The hypoglycemia risks of person-quarters exposed to DPP-4i′s and 13 concurrent medications (bumetanide, captopril, fosinopril, verapamil, simvastatin, fluvastatin, gemfibrozil, duloxetine, sulfinpyrazone, colchicine, acetaminophen, cotrimoxazole, and pantoprazole) were compared with person-quarters exposed to DPP-4i alone.

### Study Outcomes

The primary outcome was hypoglycemia diagnosed on admission or emergency visit. According to International Classification of Diseases, Ninth Revision, Clinical Modification and International Classification of Diseases, Tenth Revision, Clinical Modification diagnostic codes (Supplementary Table S1), hypoglycemia was defined as a condition that may necessitate clinical intervention involving infusion of at least two 20-ml ampules of 50% glucose or injection of 1 mg of glucagon. Patients were followed up from the first reported date of DPP-4i use to December 31, 2017, or to the date of DPP-4i discontinuation.

### Statistical Analysis

Baseline characteristics between the drug groups are reported as mean ± standard deviation and numbers with percentages. A generalized estimating equation–based Poisson model was used to estimate the adjusted prevalence of hypoglycemia in DPP-4i users between person-quarters (Twisk, 2004). Because our analytic unit was person-quarter, we computed the yearly prevalence (crude and/or adjusted) rates by applying a 0.25 weighting. Significance was set at *p* < 0.05. All analyses were performed using SAS software, version 9.4. To validate our findings and assess potential selection bias, we performed a sensitivity analysis using a negative control outcome unrelated to hypoglycemia; specifically, we used cataract operation for analysis to assess the pattern of analysis.

## Results

### Study Population

Data on 97,227 patients with T2DM taking DPP-4i′s were retrieved from the CGRD for the period 2011–2017. After the exclusion of patients in accordance with the exclusion criteria, 77,047 patients remained for analysis, corresponding to a total of 694,300 person-quarters (Figure 1; Table 1). The mean age of the patients was 64 ± 12 years, with the Charlson comorbidity index being 3.39 ± 2.33. Patients with comorbidities of hypertension, myocardial infarction, congestive heart failure, peripheral vascular disease, and cerebrovascular disease constituted 71.4, 4.7, 10.23, 3.45, and 21.09% of the study population, respectively. Regarding the commonly coprescribed ADMs, biguanide, sulfonylurea, alpha-glucosidase inhibitors, thiazolidinedione, meglitinide, and sodium-glucose cotransporter two inhibitors were prescribed in 75.96, 53.09, 13.19, 10.32, 6.23, and 0.39% of the study population, respectively.

**TABLE 1:** Baseline characteristics of study patients.

	DPP-4i users		Sitagliptin users		Saxagliptin users		Linagliptin users		Vildagliptin users		Alogliptin users	
	(*n* = 77,047)		(*n* = 38,732)		(*n* = 13,624)		(*n* = 12,973)		(*n* = 11,223)		(*n* = 495)	
Age (years)	64	±12	64	±12	62	±12	66	±13	63	±12	62	±12
Men	41,928	54.42%	20,775	53.64%	7557	55.47%	7316	56.39%	6035	53.77%	245	49.49%
Charlson comorbidity index	3.39	±2.33	3.41	±2.28	3.15	±2.26	3.79	±2.59	3.16	±2.18	2.78	±2.20
Cardiovascular diseases												
Hypertension	55,067	71.47%	28,269	72.99%	9304	68.29%	9612	74.09%	7569	67.44%	313	63.23%
Myocardial infarction	3623	4.70%	1929	4.98%	641	4.70%	675	5.20%	366	3.26%	12	2.42%
Congestive heart failure	7881	10.23%	4175	10.78%	1240	9.10%	1636	12.61%	804	7.16%	26	5.25%
Peripheral vascular disease	2657	3.45%	1355	3.50%	425	3.12%	551	4.25%	319	2.84%	7	1.41%
Percutaneous coronary intervention	4241	5.50%	2317	5.98%	746	5.48%	748	5.77%	417	3.72%	13	2.63%
Coronary artery bypass surgery	558	0.72%	279	0.72%	83	0.61%	118	0.91%	75	0.67%	3	0.61%
Diseases of the nervous system												
Cerebrovascular disease	16,252	21.09%	8901	22.98%	2340	17.18%	2863	22.07%	2106	18.77%	42	8.48%
Ischemic stroke	12,224	15.87%	6931	17.89%	1751	12.85%	2031	15.66%	1485	13.23%	26	5.25%
Transient ischemic attack	1625	2.11%	846	2.18%	244	1.79%	324	2.50%	211	1.88%	0	0.00%
Hemiplegia and paraplegia	1272	1.65%	655	1.69%	187	1.37%	211	1.63%	214	1.91%	5	1.01%
Dementia	2847	3.70%	1409	3.64%	415	3.05%	600	4.62%	410	3.65%	13	2.63%
Metabolic disease												
Diabetes mellitus	71,524	92.83%	36,852	95.15%	12,750	93.58%	11,358	87.55%	10,164	90.56%	400	80.81%
Diabetes with complications	22,688	29.45%	11,667	30.12%	3504	25.72%	4079	31.44%	3292	29.33%	146	29.49%
Pulmonary disease												
Chronic pulmonary disease	12,755	16.55%	6478	16.73%	2190	16.07%	2220	17.11%	1794	15.99%	73	14.75%
Chronic obstructive pulmonary disease	10,315	13.39%	5188	13.39%	1697	12.46%	1907	14.70%	1463	13.04%	60	12.12%
Chronic kidney disease	16,013	20.78%	7455	19.25%	2157	15.83%	4501	34.70%	1828	16.29%	72	14.55%
Gastrointestinal and hepatic diseases												
Peptic ulcer disease	20,917	27.15%	10,723	27.69%	3597	26.40%	3619	27.90%	2853	25.42%	125	25.25%
Mild liver disease	20,624	26.77%	10,369	26.77%	3813	27.99%	3358	25.88%	2945	26.24%	139	28.08%
Moderate or severe liver disease	698	0.91%	350	0.90%	105	0.77%	170	1.31%	70	0.62%	3	0.61%
Miscellaneous diseases												
Malignancy, including leukemia/lymphoma	7938	10.30%	3769	9.73%	1370	10.06%	1668	12.86%	1092	9.73%	39	7.88%
Metastatic tumor	1132	1.47%	525	1.36%	193	1.42%	269	2.07%	139	1.24%	6	1.21%
Human Immunodeficiency virus infection	18	0.02%	4	0.01%	3	0.02%	5	0.04%	5	0.04%	1	0.20%
Medication used												
Sulfonylurea	40,907	53.09%	22,024	56.86%	7394	54.27%	6126	47.22%	5100	45.44%	263	53.13%
Biguanide	58,528	75.96%	28,658	73.99%	11,345	83.27%	8088	62.34%	9989	89.00%	448	90.51%
Thiazolidinedione	7948	10.32%	5118	13.21%	1119	8.21%	843	6.50%	798	7.11%	70	14.14%
Sodium-glucose cotransporter 2 inhibitor	298	0.39%	74	0.19%	36	0.26%	119	0.92%	39	0.35%	30	6.06%
Meglitinide	4802	6.23%	2489	6.43%	700	5.14%	1071	8.26%	526	4.69%	16	3.23%
α-Glucosidase Inhibitor	10,159	13.19%	5999	15.49%	1582	11.61%	1545	11.91%	978	8.71%	55	11.11%

### Risk of Hypoglycemia With Concurrent use of Specific Medications

**FIGURE 2:**
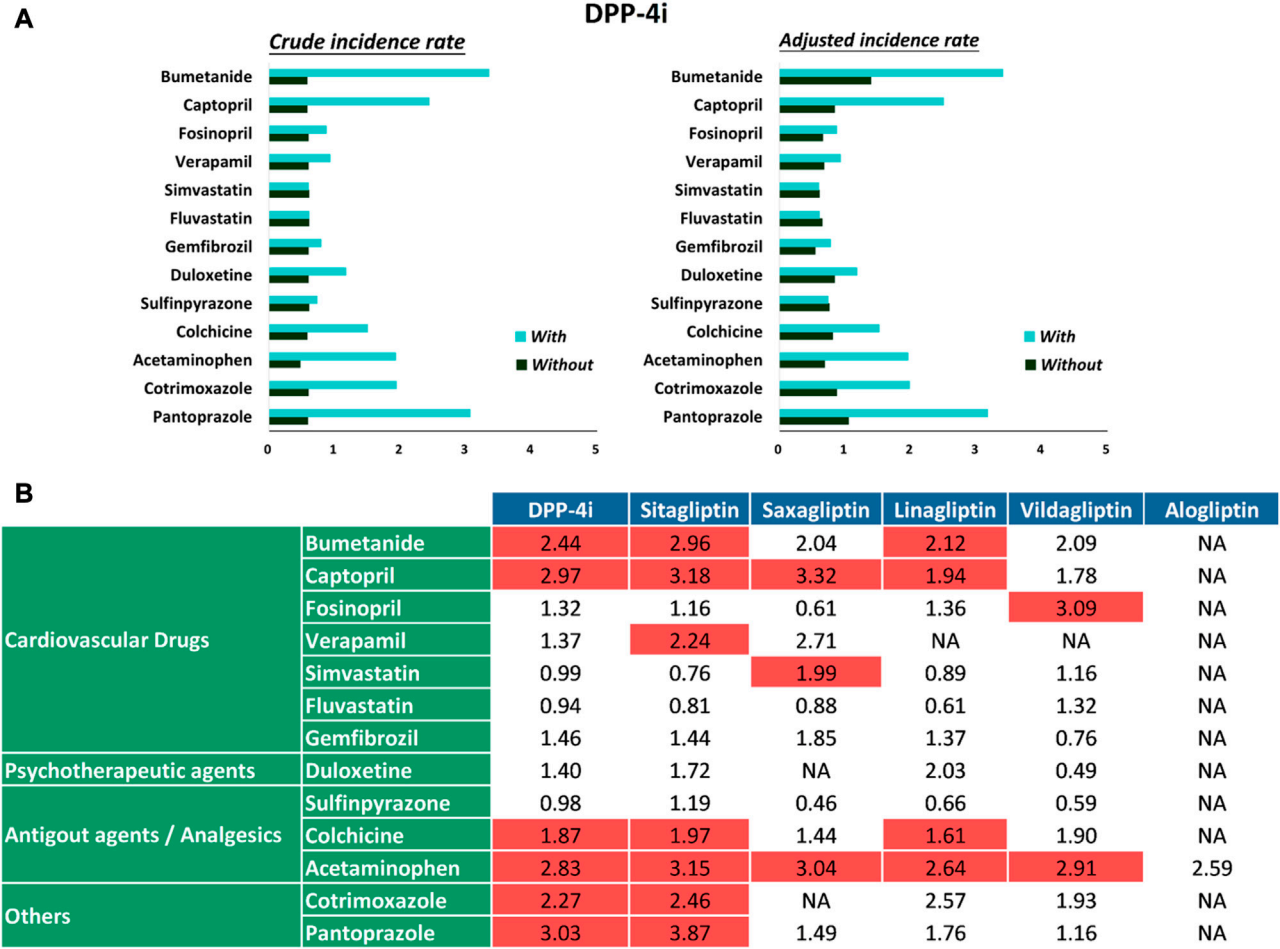
**(A)** Crude prevalence and adjusted prevalence of hypoglycemia associated with drug–drug interactions between DPP-4i′s and selected medications. **(B)** Adjusted prevalence ratios of hypoglycemia associated with drug–drug interactions between DPP-4i′s and selected medications.

During the follow-up period, 13,546 hypoglycemia events occurred in 694,300 person-quarters with DPP-4i prescriptions. Table 2 and Figure 2A present a summary of the prevalence, adjusted prevalence, and adjusted prevalence differences for hypoglycemia associated with drug–drug interactions between the DPP-4i′s and 13 concurrent medications. Supplementary Figures S1–S5 illustrate the crude and adjusted prevalence for hypoglycemia associated with drug–drug interactions between individual DPP-4i′s (sitagliptin, saxagliptin, linagliptin, vildagliptin, and alogliptin) and the selected medications. The most common medications coprescribed with DPP-4i′s over all person-quarters were acetaminophen (65,188 person-quarters), simvastatin (46,989 person-quarters), fluvastatin (30,318 person-quarters), and colchicine (22,006 person-quarters) (all >20,000 person-quarters). Combinations of a DPP-4i with bumetanide, captopril, acetaminophen, and pantoprazole were associated with an increased risk of hypoglycemia. Compared with the ratios observed for person-quarters of DPP-4i use alone (reference category), those with greater than twice (>2-fold) the adjusted rate ratios per 100 person-years of hypoglycemia for person-quarters of DPP-4i use in combination with bumetanide, captopril, colchicine, acetaminophen, cotrimoxazole, and pantoprazole were 2.44 (95% confidence interval [CI], 1.78–3.36), 2.97 (95% CI, 2.26–3.90), 2.83 (95% CI, 2.44–3.29), 2.27 (95% CI, 1.27–4.04), 3.03 (95% CI, 1.96–4.68), respectively. Combined use of a DPP-4i with the other medications was not associated with increased prevalence of hypoglycemia. Colchicine was associated with a reduced adjusted prevalence of hypoglycemia. Figure 2B displays the adjusted prevalence ratio, and Supplementary Table S2 presents the results obtained from the analysis of the negative control outcome. We observed a high prevalence of cataract operation in patients concomitantly using a DPP-4i and acetaminophen; this can probably be attributed to the high rates of analgesic prescriptions in patients undergoing cataract surgery.

**TABLE 2:** Selected drugs to study for drug-drug interactions.

Concurrent medication		Person-quarters with DPP-4i	No. of events	Crude Incidence Rate (95% CI) per 100 Person-Years		Adjusted Incidence Rate (95% CI) per 100 Person-Years		Adjusted Rate Ratio (95% CI)		P value	Adjusted Incidence Rate Difference (95% CI) per 100 Person-Years		P value
Bumetanide	With	6298	53	3.35	(2.51−4.48)	3.40	(2.56−4.52)	2.44	(1.78−3.36)	<0.0001	2.01	(1.02- 2.99)	<0.0001
Bumetanide	Without	688002	989	0.58	(0.54−0.62)	1.39	(1.21−1.60)	1	(1.00−1.00)				
Captopril	With	9419	58	2.44	(1.87−3.20)	2.50	(1.92−3.25)	2.97	(2.26−3.90)	<0.0001	1.66	(1.00- 2.32)	<0.0001
Captopril	Without	684881	984	0.58	(0.54−0.62)	0.84	(0.77−0.92)	1	(1.00−1.00)				
Fosinopril	With	9292	20	0.87	(0.55−1.37)	0.87	(0.55−1.37)	1.32	(0.83−2.10)	0.2375	0.21	(−0.19−0.61)	0.2994
Fosinopril	Without	685008	1022	0.60	(0.56−0.64)	0.66	(0.60−0.71)	1	(1.00−1.00)				
Verapamil	With	6441	15	0.93	(0.54−1.58)	0.93	(0.54−1.58)	1.37	(0.80−2.34)	0.2585	0.25	(−0.25−0.75)	0.3286
Verapamil	Without	687859	1027	0.60	(0.57−0.65)	0.68	(0.62−0.74)	1	(1.00−1.00)				
Simvastatin	With	46,989	70	0.60	(0.46−0.79)	0.60	(0.46−0.79)	0.99	(0.75−1.31)	0.9377	−0.01	(−0.18−0.16)	0.9374
Simvastatin	Without	647311	972	0.61	(0.57−0.65)	0.61	(0.57−0.66)	1	(1.00−1.00)				
Fluvastatin	With	30,318	46	0.61	(0.45−0.84)	0.61	(0.45−0.83)	0.94	(0.69−1.30)	0.7269	−0.04	(−0.23−0.16)	0.7202
Fluvastatin	Without	663982	996	0.61	(0.57−0.65)	0.65	(0.60−0.70)	1	(1.00−1.00)				
Gemfibrozil	With	16,375	32	0.79	(0.53−1.16)	0.78	(0.53−1.16)	1.46	(0.97−2.18)	0.0681	0.25	(-0.07−0.56)	0.1238
Gemfibrozil	Without	677925	1010	0.60	(0.56−0.64)	0.54	(0.49−0.59)	1	(1.00−1.00)				
Duloxetine	With	4483	13	1.17	(0.67−2.05)	1.18	(0.68−2.04)	1.4	(0.80−2.45)	0.2321	0.34	(-0.31−0.99)	0.3069
Duloxetine	Without	689817	1029	0.60	(0.56−0.64)	0.84	(0.76−0.93)	1	(1.00−1.00)				
Sulfinpyrazone	With	8796	16	0.73	(0.41−1.30)	0.74	(0.42−1.31)	0.98	(0.55−1.74)	0.9386	−0.02	(−0.44−0.41)	0.9379
Sulfinpyrazone	Without	685504	1026	0.61	(0.57−0.65)	0.76	(0.70−0.82)	1	(1.00−1.00)				
Colchicine	With	22,006	82	1.50	(1.17−1.92)	1.52	(1.19−1.94)	1.87	(1.44−2.42)	<0.0001	0.70	(0.32−1.08)	0.0003
Colchicine	Without	672294	960	0.58	(0.54−0.62)	0.81	(0.74−0.89)	1	(1.00−1.00)				
Acetaminophen	With	65,188	319	1.93	(1.73−2.16)	1.96	(1.75−2.19)	2.83	(2.44−3.29)	<0.0001	1.27	(1.04−1.50)	<0.0001
Acetaminophen	Without	629112	723	0.47	(0.43−0.50)	0.69	(0.62−0.77)	1	(1.00−1.00)				
Cotrimoxazole	With	2443	12	1.94	(1.09−3.47)	1.98	(1.12−3.49)	2.27	(1.27−4.04)	0.0054	1.11	(−0.02−2.23)	0.054
Cotrimoxazole	Without	691857	1030	0.60	(0.56−0.64)	0.87	(0.78−0.98)	1	(1.00−1.00)				
Pantoprazole	With	3346	26	3.06	(2.00−4.69)	3.17	(2.10−4.79)	3.03	(1.96−4.68)	<0.0001	2.12	(0.81−3.44)	0.0016
Pantoprazole	Without	690954	1016	0.59	(0.56−0.64)	1.05	(0.92−1.19)	1	(1.00−1.00)				

## Discussion

To the best of our knowledge, our study is the first to examine a wide range of commonly prescribed medications and their drug–drug interactions with DPP-4i′s (which among the safest categories of ADMs) (Idris andand Donnelly, 2007). Our findings suggest that when used in combination with DPP-4i′s, bumetanide, captopril, acetaminophen, cotrimoxazole, and pantoprazole may increase the risk of hypoglycemia more than twice and require the intervention of glucose infusion or glucagon injection.

DPP-4i′s have favorable pharmacokinetic characteristics, are neither inducers nor inhibitors of cytochrome P-450 isoforms, and have few or no reported drug interactions (Idris andand Donnelly, 2007). They do not significantly modify the pharmacokinetic profile and exposure of other drugs; similarly, other drugs do not significantly alter the pharmacokinetic profile of DPP-4i′s (Scheen, 2005; Tella and Rendell, 2015). However, drugs commonly coprescribed to patients with T2DM, such as statins and antihypertensive agents, interfere with the cytochrome (CYP) P-450 system (Scheen, 2005; Idris and Donnelly, 2007; Tella and Rendell, 2015). Although the mechanism of action of all DPP-4 inhibitors are considered similar, slightly different pharmacokinetic properties have been described. Saxagliptin is metabolized via CYP3A4/5, and thus can interact with CYP3A4-inhibitors (e.g., ketoconazole, diltiazem, ritonavir and clarithromycin) or CYP3A4-inductors (e.g., rifampicin) (Filippatos et al., 2014; Tella and Rendell, 2015). However, other DPP-4i′s such as sitagliptin, vildagliptin, linagliptin, and aloptin are either being less of a substrate or not as a substrate for CYP3A4/5 (Capuano et al., 2013). In addition, a high percentage of certain DPP-4i′s such as sitagliptin, viltagliptin, saxagliptin, and alogliptin are renally excreted (75–87%)except linagliptin (5%) (Capuano et al., 2013).

Although drug–drug interactions between DPP-4i′s and other drugs have been reported to be rare in preclinical studies, it is imperative such evidences from large-scale, real-world population-based research also observed. T2DM patients with cardiovascular disease and elderly T2DM patients having multiple prescriptions are especially at a high risk of drug-drug interactions. Studies of drug-drug interactions between DPP-4i′s and concurrent medications have only been investigated in diuretics, ACE inhibitors, calcium channel blockers, statins, antibiotics, and proton pump inhibitors in terms of translational researches (Kim et al., 2006; Ayalasomayajula et al., 2007; Chu et al., 2007; He et al., 2008; Patel et al., 2011; Patel et al., 2011; Arulmozhiraja et al., 2016; May and Schindler, 2016). In this study, we explored possible drug–drug interactions between DPP4i′s and concurrent medications that cause hypoglycemic events requiring clinical intervention. Our results show that the commonly prescribed cardiovascular medications bumetanide and captopril, the analgesic acetaminophen, and the proton pump inhibitor pantoprazole may cause sufficiently severe hypoglycemia necessitating glucose infusion or glucagon injection at a hospital. For instances, bumetanide may cause increased incidence of hypoglycemia when prescribed with sitagliptin and linagliptin. Captopril may cause increased incidence of hypoglycemia when prescribed with sitagliptin, saxagliptin, and linagliptin. Colchicine may cause increased incidence of hypoglycemia with prescribed with sitagliptin and linagliptin. Acetaminophen may cause increased incidence of hypoglycemia when prescribed with sitagliptin, saxagliptin, linagliptin, and vildagliptin. Pantoprazole may cause increased incidence of hypoglycemia when prescribed with sitagliptin, may due in part that decreased renal excretion of sitagliptin via transporter OAT3 when coadministered with OAT3 inhibitors such as pantoprazole (Chu et al., 2007; Wang et al., 2019). Notably, although ACE inhibitors are generally safe medications (Marney et al., 2010), captopril was observed to be associated with hypoglycemia events when used in addition to DPP-4i′s. In contrast to previous research suggesting interactions between the nondihydropyridine calcium channel blocker diltiazem and a DPP-4i (He et al., 2008), we observed that verapamil did not have adverse drug–drug interactions that could lead to hypoglycemia. In particular, a frequently prescribed analgesic such as acetaminophen may not be as safe as previously thought when taken with a DPP-4i. However, additional studies are required to determine whether this phenomenon can be observed in other ethnic populations subjected to the same coprescription. Our negative control analysis using cataract operation as an outcome revealed a high prevalence of cataracts in patients concomitantly using DPP-4i′s and acetaminophen; this was probably associated with their use of analgesics.

In summary, this is the first nationwide population-based study on the drug–drug interactions between DPP-4i and concurrent medications to measure the risk of hypoglycemia. Postmarketing surveillance using large patient registries should be helpful to improve the detection of clinically relevant DPP-4i drug–drug interactions.

### Limitations

Several limitations are associated with the use of epidemiological data from the CGRD. First, the use of *International Classification of Diseases, Ninth Revision, Clinical Modification* and *International Classification of Diseases, Tenth Revision, Clinical Modification* codes for patient screening may have resulted in missing cases for conditions coded incorrectly. Second, the data from the claim-based CGRD had inherently limited clinical medical information, such as examination report details. Third, alogliptin had a relatively late introduction in Taiwan, and thus the number of patients taking alogliptin (in the hundreds) was relatively small compared with that of those taking other DPP-4i′s (in the thousands). Therefore, many drug–drug interactions could not be analyzed. Fourth, because this study involved a retrospective and observational design, causality could not be established. Finally, the study sample and background population were ethnically homogenous; therefore, the generalizability of the results to other populations and settings may require further research.

## Conclusion

Among patients taking DPP-4i′s for T2DM, concurrent use of such inhibitors with bumetanide, captopril, acetaminophen, cotrimoxazole, and pantoprazole was associated with an increased risk of hypoglycemia compared with the use of DPP-4i′s alone. Physicians prescribing DPP-4i′s should consider the potential risks associated with concomitant use of the inhibitors with other drugs.

## Data Availability

The original contributions presented in the study are included in the article/Supplementary Material, further inquiries can be directed to the corresponding author.
