# Supplementary material for: Hypoglycemia Associated With Drug–Drug Interactions in Patients With Type 2 Diabetes Mellitus Using Dipeptidylpeptidase-4 Inhibitors
Source: Front Pharmacol. 2021 Apr 15;12:570835. doi: 10.3389/fphar.2021.570835 (PMC8142266; doi:10.3389/fphar.2021.570835)
Supplement: Supplementary file 1 [file datasheet1.docx]

**Supplementary Appendix**

**Supplementary Table 1.** ICD codes

**Supplementary Figure 1.** Sitagliptin drug-drug interactions with crude prevalence rate and adjusted prevalence rate.

**Supplementary Figure 2.** Saxagliptin drug-drug interactions with crude prevalence rate and adjusted prevalence rate.

**Supplementary Figure 3.** Lingliptin drug-drug interactions with crude prevalence rate and adjusted prevalence rate.

**Supplementary Figure 4.** Vildagliptin drug-drug interactions with crude prevalence rate and adjusted prevalence rate.

**Supplementary Figure 5.** Alogliptin drug-drug interactions with crude prevalence rate and adjusted prevalence rate.

**Supplementary Table 2.** Negative control events and incidences using cataract operation

**Supplementary Table 1.** ICD codes

| Disease | ICD-9-CM | ICD-10-CM |
| --- | --- | --- |
| Hypoglycemia | 2510, 2511, 2512, 25030, 25080, 24930, 24980, 25000 | E15, E160, E161, E162, E1164, E1364, E0964, E0864 |
| Insulinoma | 157, 2117 | C25, D137 |
| Hypertension | 401, 402 | I10-I16 |
| Myocardial infarction | 410.x, 412.x | I21.x, I22.x, I25.2 |
| Congestive heart failure | 398.91, 402.01, 402.11, 402.91,404.01, 404.03, 404.11, 404.13, 404.91, 404.93, 425.4–425.9, 428.x | I09.9, I11.0, I13.0, I13.2, I25.5, I42.0, I42.5–I42.9, I43.x, I50.x, P29.0 |
| Peripheral vascular disease | 093.0, 437.3, 440.x, 441.x, 443.1–443.9, 557.1, 557.9, V43.4 | I70.x, I71.x, I73.1, I73.8, I73.9, I77.1, I79.0, I79.2, K55.1, K55.8, K55.9, Z95.8, Z95.9 |
| Cerebrovascular disease | 362.34, 430.x–438.x | G45.x, G46.x, H34.0, I60.x–I69.x |
| Ischemic stroke | 433, 434, 436, 438, 4371 | I63, I67.81, I67.82, G46 |
| Transient ischemic attack | 435 | G45 |
| Hemiplegia and paraplegia | 334.1, 342.x, 343.x, 344.0– 344.6, 344.9 | G04.1, G11.4, G80.1, G80.2, G81.x, G82.x, G83.0–G83.4, G83.9 |
| Dementia | 290.x, 294.1, 331.2 | F00.x–F03.x, F05.1, G30.x, G31.1 |
| Diabetes mellitus | 250 | E10.0; E10.1; E10.9; E11.0; E11.1; E11.9 |
| Diabetes with complications | 250.4–250.7 | E10.2–E10.5, E10.7, E11.2–E11.5, E11.7, E12.2–E12.5, E12.7, E13.2– E13.5, E13.7, E14.2–E14.5, E14.7 |
| Chronic pulmonary disease | 416.8, 416.9, 490.x–505.x, 506.4, 508.1, 508.8 | I27.8, I27.9, J40.x–J47.x, J60.x–J67.x, J68.4, J70.1, J70.3 |
| Chronic obstructive pulmonary disease | 490, 491.0, 491.1, 491.2, 491.20, 491.21, 491.22, 491.8, 491.9, 492.0, 492.8, 494, 494.0, 494.1, 496 | J40, J41.0, J41.1, J41.8, J42, J43.0, J43.1, J43.2, J43.8, J43.9, J44.0, J44.1, J44.9, J47.0, J47.1, J47.9 |
| Chronic kidney disease | 580, 581, 582, 583, 584, 585, 586, 587, 588, 589 | I12 I13 N00-N05 N07 N11 N14 N17 N18 N19 Q61 |
| Peptic ulcer disease | 531.x–534.x | K25.x–K28.x |
| Mild liver disease | 070.22, 070.23, 070.32, 070.33, 070.44, 070.54, 070.6, 070.9, 570.x, 571.x, 573.3, 573.4, 573.8, 573.9 | B18.x, K70.0–K70.3, K70.9, K71.3–K71.5, K71.7, K73.x, K74.x, K76.0, K76.2–K76.4, K76.8, K76.9, Z94.4 |
| Moderate or severe liver disease | 456.0–456.2, 572.2–572.8 | I85.0, I85.9, I86.4, I98.2, K70.4, K71.1, K72.1, K72.9, K76.5, K76.6, K76.7 |
| Any malignancy, including leukemia and lymphoma | 140.x–172.x, 174.x–195.8, 200.x–208.x, 238.6 | C00.x–C26.x, C30.x–C34.x, C37.x–C41.x, C43.x, C45.x–C58.x, C60.x–C76.x, C81.x–C85.x, C88.x, C90.x–C97.x |
| Metastatic tumor | 196.x–199.x | C77.x–C80.x |
| Human Immunodeficiency virus | 042.x–044.x | B20.x–B22.x, B24.x |

**Supplementary Figure 1.** Sitagliptin drug-drug interactions with crude prevalence rate and adjusted prevalence rate.

**
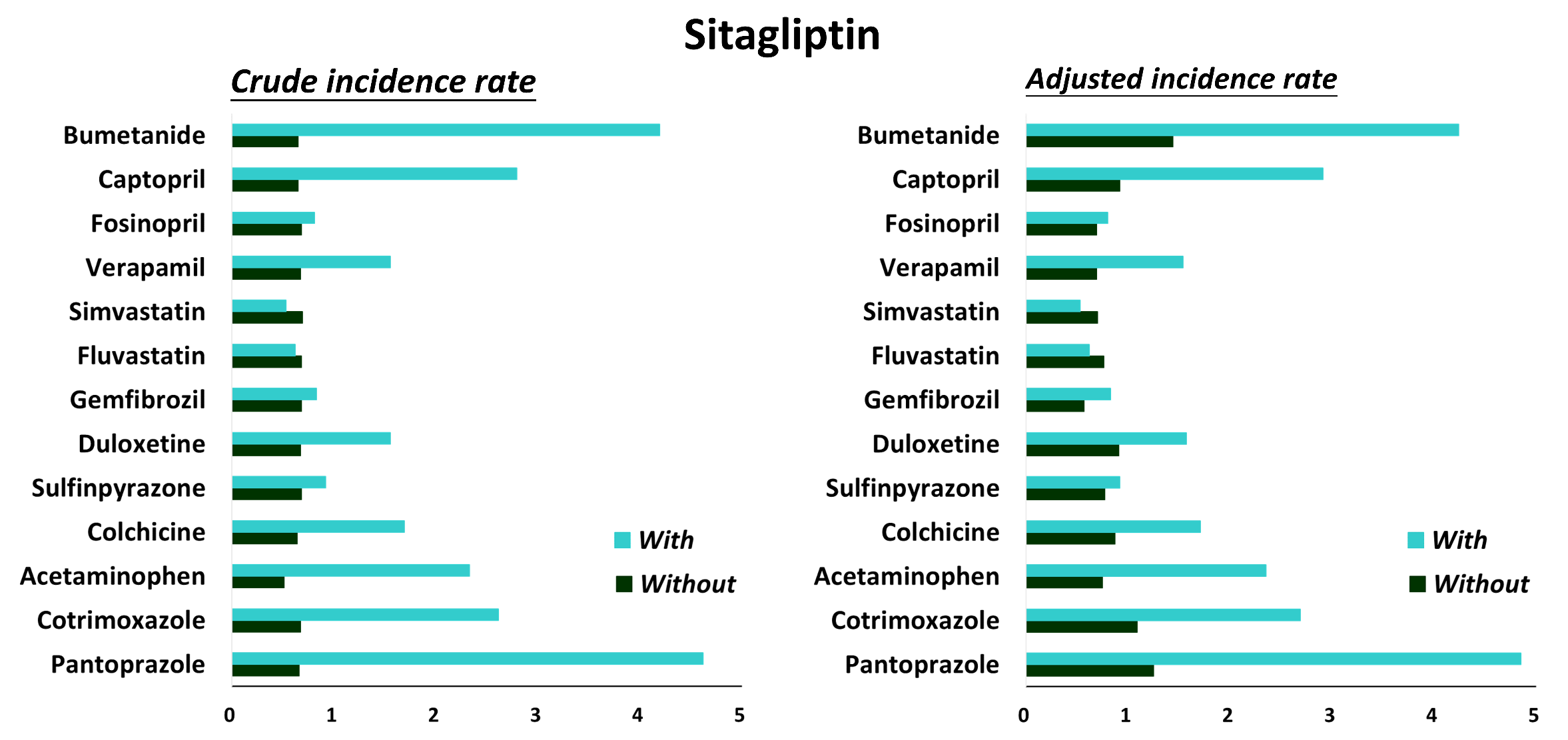
**

**Supplementary Figure 2.** Saxagliptin drug-drug interactions with crude prevalence rate and adjusted prevalence rate.

**
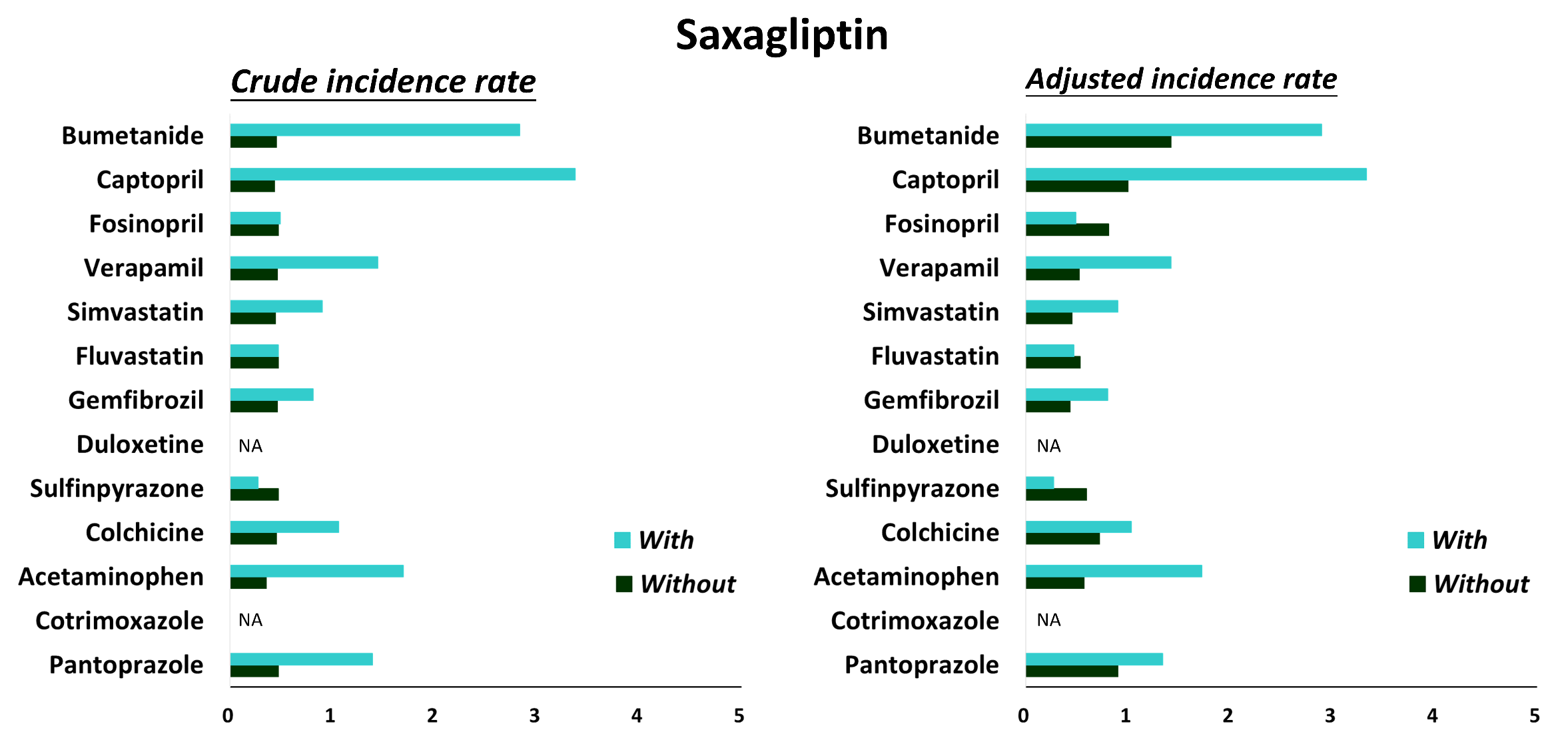
**

**Supplementary Figure 3.** Linagliptin drug-drug interactions with crude prevalence rate and adjusted prevalence rate.

**
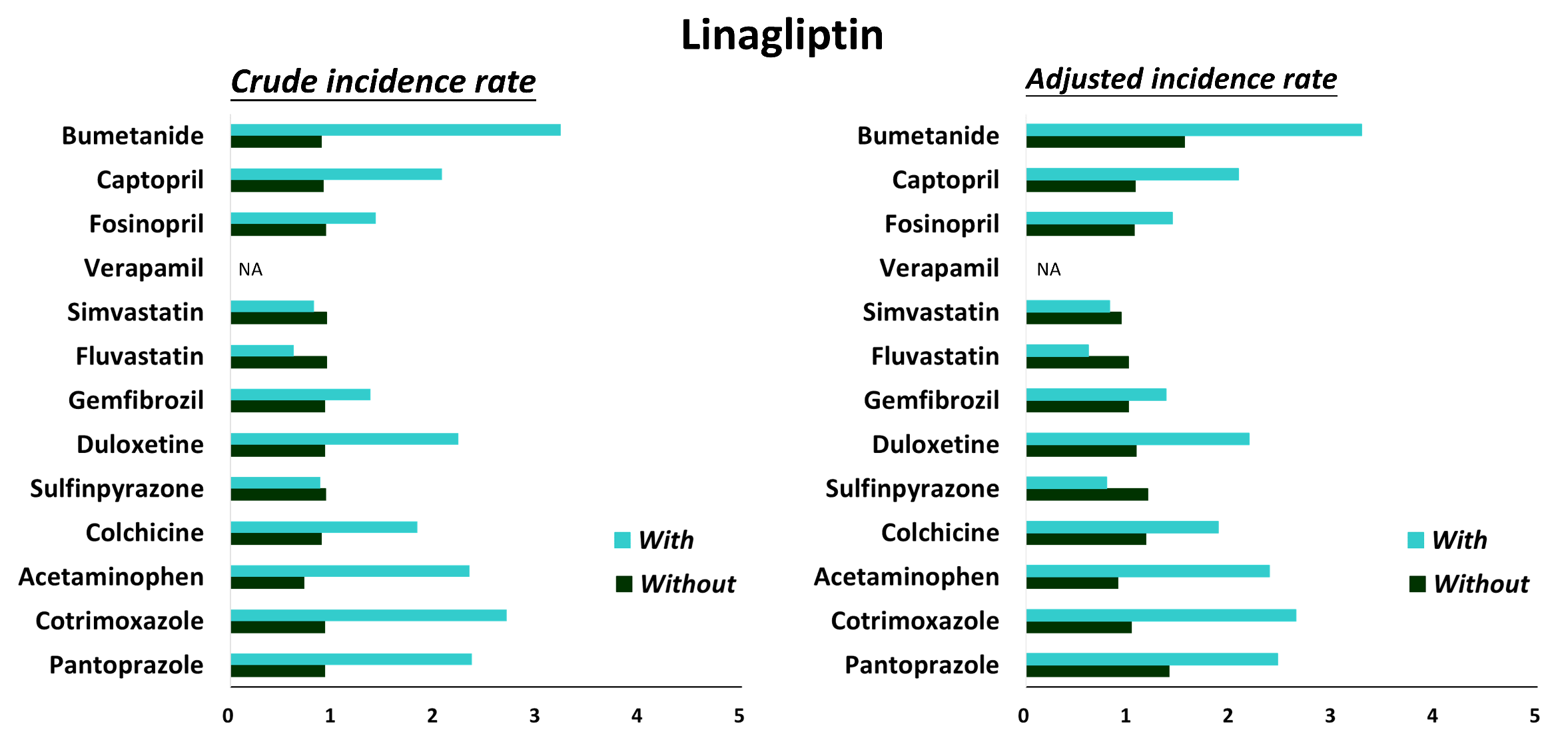
**

**Supplementary Figure 4.** Vildagliptin drug-drug interactions with crude prevalence rate and adjusted prevalence rate.

**
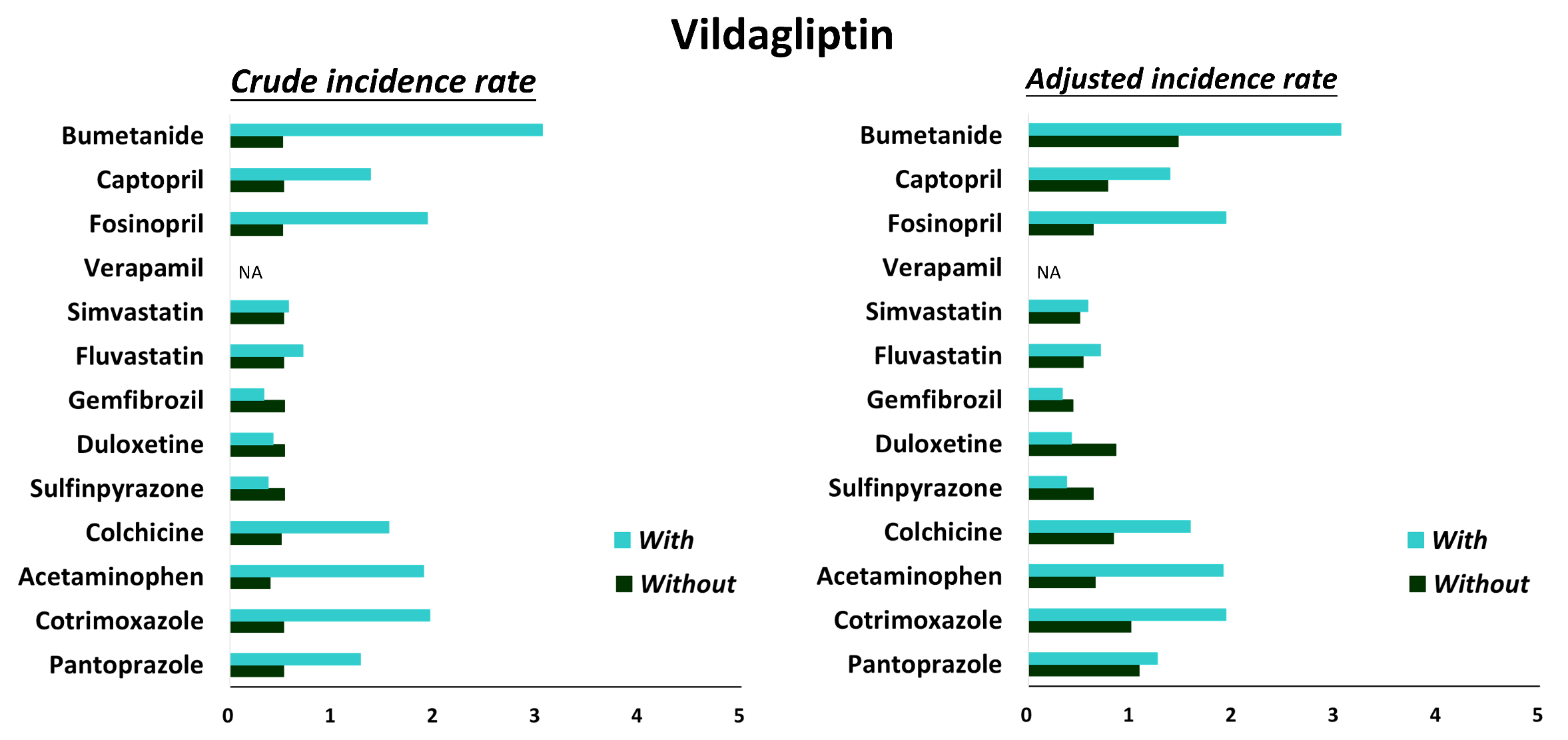
**

**Supplementary Figure 5.** Alogliptin drug-drug interactions with crude prevalence rate and adjusted prevalence rate.

**
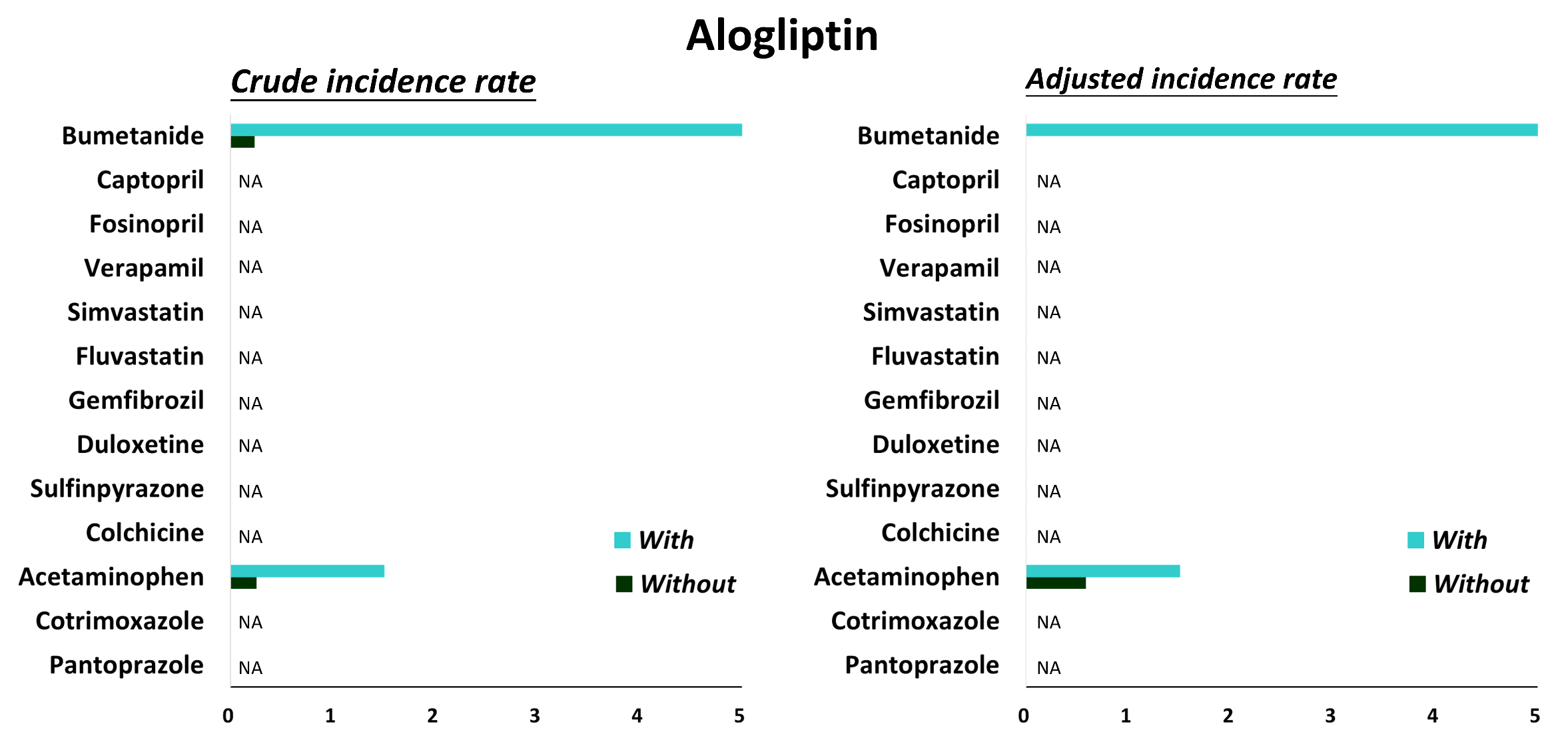
**

**Supplementary Table 2.** Negative control events and incidences using cataract operation

|  | **Concurrent medication** | | **Person-Quarters with DPP-4i use** | **No. of Events** | **Crude Incidence Rate (95% CI) per 100 Person-Years** | | **Adjusted Incidence Rate (95% CI) per 100 Person-Years** | | **Adjusted Rate Ratio (95% CI)** | | **P value** | **Adjusted Incidence Rate Difference (95% CI) per 100 Person-Years** | | **P value** |
| --- | --- | --- | --- | --- | --- | --- | --- | --- | --- | --- | --- | --- | --- | --- |
| 2 | Bumetanide | With | 6306 | 25 | 1.53 | ( 1.00- 2.34) | 1.57 | ( 1.03- 2.38) | 0.93 | ( 0.61 - 1.44) | 0.7571 | -0.11 | ( -0.79- 0.57) | 0.7501 |
| 2 | Bumetanide | Without | 687994 | 2035 | 1.18 | ( 1.12- 1.24) | 1.68 | ( 1.51- 1.87) | 1 | ( 1.00 - 1.00) | . | . |  | . |
| 3 | Captopril | With | 9438 | 30 | 1.21 | ( 0.83- 1.78) | 1.25 | ( 0.86- 1.82) | 0.97 | ( 0.66 - 1.41) | 0.8595 | -0.04 | ( -0.52- 0.43) | 0.8572 |
| 3 | Captopril | Without | 684862 | 2030 | 1.18 | ( 1.12- 1.24) | 1.3 | ( 1.22- 1.38) | 1 | ( 1.00 - 1.00) | . | . |  | . |
| 4 | Fosinopril | With | 9292 | 21 | 0.91 | ( 0.56- 1.47) | 0.91 | ( 0.56- 1.46) | 0.71 | ( 0.44 - 1.15) | 0.1677 | -0.37 | ( -0.81- 0.07) | 0.1030 |
| 4 | Fosinopril | Without | 685008 | 2039 | 1.18 | ( 1.13- 1.24) | 1.27 | ( 1.20- 1.35) | 1 | ( 1.00 - 1.00) | . | . |  | . |
| 6 | Verapamil | With | 6440 | 26 | 1.59 | ( 1.03- 2.45) | 1.61 | ( 1.04- 2.47) | 1.18 | ( 0.76 - 1.83) | 0.4500 | 0.25 | ( -0.45- 0.95) | 0.4854 |
| 6 | Verapamil | Without | 687860 | 2034 | 1.18 | ( 1.12- 1.24) | 1.36 | ( 1.27- 1.45) | 1 | ( 1.00 - 1.00) | . | . |  | . |
| 7 | Simvastatin | With | 46988 | 118 | 0.99 | ( 0.81- 1.20) | 0.99 | ( 0.81- 1.21) | 0.85 | ( 0.69 - 1.04) | 0.1106 | -0.18 | ( -0.39- 0.03) | 0.0864 |
| 7 | Simvastatin | Without | 647312 | 1942 | 1.19 | ( 1.13- 1.26) | 1.17 | ( 1.11- 1.24) | 1 | ( 1.00 - 1.00) | . | . |  | . |
| 9 | Fluvastatin | With | 30318 | 74 | 0.96 | ( 0.75- 1.24) | 0.97 | ( 0.75- 1.25) | 0.79 | ( 0.61 - 1.02) | 0.0708 | -0.26 | ( -0.52- -0.01) | 0.0438 |
| 9 | Fluvastatin | Without | 663982 | 1986 | 1.19 | ( 1.13- 1.25) | 1.23 | ( 1.16- 1.29) | 1 | ( 1.00 - 1.00) | . | . |  | . |
| 10 | Gemfibrozil | With | 16372 | 56 | 1.37 | ( 1.02- 1.83) | 1.37 | ( 1.02- 1.83) | 1.13 | ( 0.84 - 1.52) | 0.4355 | 0.15 | ( -0.25- 0.56) | 0.4603 |
| 10 | Gemfibrozil | Without | 677928 | 2004 | 1.17 | ( 1.12- 1.23) | 1.21 | ( 1.14- 1.29) | 1 | ( 1.00 - 1.00) | . | . |  | . |
| 14 | Duloxetine | With | 4484 | 17 | 1.42 | ( 0.78- 2.60) | 1.5 | ( 0.85- 2.65) | 1.17 | ( 0.66 - 2.08) | 0.5837 | 0.22 | ( -0.63- 1.08) | 0.6119 |
| 14 | Duloxetine | Without | 689816 | 2043 | 1.18 | ( 1.12- 1.24) | 1.28 | ( 1.19- 1.37) | 1 | ( 1.00 - 1.00) | . | . |  | . |
| 16 | Sulfinpyrazone | With | 8796 | 26 | 1.15 | ( 0.72- 1.84) | 1.16 | ( 0.74- 1.84) | 0.91 | ( 0.57 - 1.44) | 0.6787 | . |  | . |
| 16 | Sulfinpyrazone | Without | 685504 | 2034 | 1.18 | ( 1.12- 1.24) | 1.28 | ( 1.20- 1.37) | 1 | ( 1.00 - 1.00) | . | . |  | . |
| 17 | Colchicine | With | 22016 | 87 | 1.55 | ( 1.22- 1.97) | 1.55 | ( 1.22- 1.97) | 1.12 | ( 0.87 - 1.44) | 0.3740 | 0.17 | ( -0.22- 0.55) | 0.3968 |
| 17 | Colchicine | Without | 672284 | 1973 | 1.17 | ( 1.11- 1.23) | 1.38 | ( 1.29- 1.49) | 1 | ( 1.00 - 1.00) | . | . |  | . |
| 18 | Acetaminophen | With | 65304 | 1935 | 12.02 | ( 11.42- 12.65) | 12.13 | ( 11.52- 12.76) | 156.58 | (122.54 -200.07) | <.0001 | 12.05 | ( 11.43- 12.66) | <.0001 |
| 18 | Acetaminophen | Without | 628996 | 125 | 0.08 | ( 0.06- 0.09) | 0.08 | ( 0.06- 0.10) | 1 | ( 1.00 - 1.00) | . | . |  | . |
| 19 | Cotrimoxazole | With | 2442 | 10 | 1.5 | ( 0.76- 2.95) | 1.59 | ( 0.82- 3.08) | 1.26 | ( 0.65 - 2.46) | 0.4884 | 0.33 | ( -0.72- 1.39) | 0.5365 |
| 19 | Cotrimoxazole | Without | 691858 | 2050 | 1.18 | ( 1.12- 1.24) | 1.25 | ( 1.17- 1.35) | 1 | ( 1.00 - 1.00) | . | . |  | . |
| 20 | Pantoprazole | With | 3357 | 11 | 1.24 | ( 0.66- 2.34) | 1.29 | ( 0.68- 2.42) | 0.89 | ( 0.47 - 1.69) | 0.7287 | -0.15 | ( -0.98- 0.67) | 0.7142 |
| 20 | Pantoprazole | Without | 690943 | 2049 | 1.18 | ( 1.12- 1.24) | 1.44 | ( 1.30- 1.59) | 1 | ( 1.00 - 1.00) | . | . |  | . |
